# Reducing the Number of Unnecessary Thyroid Nodule Biopsies With the American College of Radiology (ACR) Thyroid Imaging Reporting and Data System (TI-RADS)

**DOI:** 10.7759/cureus.23118

**Published:** 2022-03-13

**Authors:** Bader Abou Shaar, Moussa Meteb, Ghassan Awad El-Karim, Youssef Almalki

**Affiliations:** 1 Department of Radiology, Alfaisal University College of Medicine, Riyadh, SAU; 2 Department of Diagnostic Imaging, Bluewater Health, Sarnia, CAN; 3 Research Institute, The Ottawa Hospital, Ottawa, CAN; 4 Faculty of Medicine, University of Toronto, Toronto, CAN

**Keywords:** american college of radiology, thyroid imaging reporting and data system, ultrasonography, thyroid cancer, thyroid nodule, biopsy, imaging, ultrasound, nodule, thyroid

## Abstract

Introduction

Thyroid nodules are exceedingly common, occurring in up to 76% of adults. Less than 10% are palpable, and the majority are detected incidentally with an estimated prevalence of 68%, 25%, and 18% using ultrasound (US), CT, and MRI, respectively. The rising use of imaging over the last four decades has led to a significant increase in nodule detection or ‘over-identification,’ fine-needle aspiration (FNA), a higher reported incidence of thyroid cancer, and thyroidectomy. The purpose of this study is to provide a descriptive experience with thyroid nodule FNAs one year prior and one year after the implementation of the American College of Radiology (ACR) Thyroid Imaging Reporting and Data System (TI-RADS) at a prototypical community hospital.

Methods

A total of 104 patients with 114 thyroid nodules underwent US-guided FNA at Bluewater Health from January 1, 2018, to March 31, 2020, with available cytological results (The Bethesda System). The study population was divided into two cohorts (January 1, 2018, to December 31, 2018 - ‘local best practice cohort’, and March 1, 2019, to March 31, 2020 - ‘ACR TI-RADS cohort’) based on the implementation of the ACR TI-RADS guidelines in March 2019.

Results

The local best practice cohort (January 1, 2018, to December 31, 2018) comprised 57 thyroid nodules in 52 patients (mean age 66 ± 12; 40 Women). The ACR TI-RADS cohort (March 1, 2019, to March 31, 2020) comprised 57 thyroid nodules in 52 patients (mean age 61 ± 16; 41 Women). There were no statistical differences with respect to age, gender, or thyroid nodule location. Our results show a dramatic decrease in the number of unnecessary FNAs if ACR TI-RADS was implemented from January to December 2018. Thirty (52.6%) of the previously sampled thyroid nodules using the local best practice guidelines would have been followed as per ACR TI-RADS.

Conclusion

ACR TI-RADS is a reliable classification system in routine practice that significantly reduces the number of unnecessary thyroid FNAs with higher specificity compared to local best practice guidelines.

## Introduction

Thyroid nodules are exceedingly common, occurring in up to 76% of adults [1]. Less than 10% are palpable, and the majority are detected incidentally with an estimated prevalence of 68%, 25%, and 18% using ultrasound (US), CT, and MRI, respectively [1-4]. The rising use of imaging over the last four decades has led to a significant increase in nodule detection or ‘over-identification,’ fine-needle aspiration (FNA), a higher reported incidence of thyroid cancer, and thyroidectomy [1,4-5].

Papillary carcinoma is the most common malignancy, occurring in 80-90% of all cases, and has a 30-year survival rate of 95% [6]. Follicular thyroid carcinoma is the second most frequent malignancy, accounting for 10-20% of all thyroid neoplasms, and although the prognosis is not as favorable as papillary carcinoma, 10-year survival can be expected for up to 90% of patients [7]. Both of those differentiated thyroid cancers account for the greatest rise in incidence; in Canada, thyroid cancer incidence has dramatically increased by nearly five times for men and six times for women from 1970 to 2012 [8]. Despite the increasing radiological, pathological, and surgical interventions associated with thyroid nodules, low thyroid cancer mortality rates worldwide have not significantly changed [1]. 

Many risk-stratification systems were developed to address the over-diagnosis ‘epidemic,’ particularly given the associated substantial human and financial costs [9]. They include the American Thyroid Association (ATA) risk stratification system [10], Korean Society of Thyroid Radiology Thyroid Imaging Reporting and Data System (K-TIRADS) [11], American Association of Clinical Endocrinologists (AACE) [12], European Thyroid Association TIRADS (EU-TIRADS) [13], and the American College of Radiology (ACR) TI-RADS [14]. Recent studies have shown the ACR TI-RADS classification to be a reliable, non-invasive, and practical method for assessing thyroid nodules in routine practice, as well as outperforming the other classification in systems by allowing for the largest reduction of unnecessary thyroid nodule FNAs with the lowest negative predictive value at 2.2% [15-20].

The purpose of this study is to provide a descriptive experience with thyroid nodule FNAs one year prior to and one year after the implementation of the ACR TI-RADS at a prototypical community hospital.

## Materials and methods

Study setting

Bluewater Health (BWH) is a dual-site secondary care center with 330 beds serving the county of Sarnia-Lambton, Ontario, Canada, with a catchment area of 150,000 people. Prior to the implementation of the ACR TI-RADS stratification system in March 2019, US-guided thyroid nodule FNAs were performed according to local best practice (LBP) inspired by the ATA guidelines. While the sonographic suspicion categories were the same as outlined in the ATA guidelines, the size criteria for biopsy were more conservative (e.g., local best practice recommends FNA at >2 cm for an intermediate sonographic pattern compared to ATA recommendations at >1 cm) [10,21].

The research ethics board approved this study, and the principles of the Declaration of Helsinki were followed. Informed signed consent was not required.

Study population

**Figure 1:**
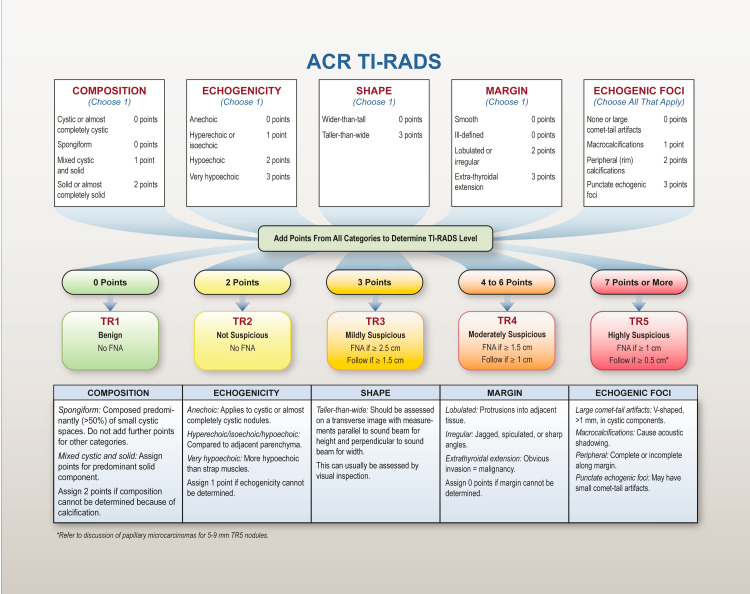
American College of Radiology Thyroid Imaging Reporting and Data System risk stratification system and management recommendations ACR TI-RADS = American College of Radiology Thyroid Imaging Reporting and Data System; FNA = fine-needle aspiration. Adapted from the White Paper of the ACR TI-RADS committee [23]

A total of 104 patients with 114 thyroid nodules underwent US-guided FNA at Bluewater Health from January 1, 2018, to March 31, 2020, with available cytological results (The Bethesda System) [22]. Patients with incomplete clinical information and, or absent cytological results were excluded from the analysis. The study population was divided into two cohorts (January 1, 2018, to December 31, 2018 - ‘Local Best Practice cohort’, and March 1, 2019, to March 31, 2020 - ‘ACR TI-RADS cohort’) based on the implementation of the ACR TI-RADS guidelines in March 2019 (Figure 1).

Image interpretation and ACR TI-RADS categorization

A total of eight radiologists with between two and 20 years of post-training experience routinely interpret thyroid US exams at Bluewater Health. Recommendations for thyroid nodule FNA prior to the implementation of the ACR TI-RADS criteria were based on local best practice guidelines motivated by ATA guidelines (Table 1), as discussed previously [10,21].

**Table 1:** Comparison of ATA and local best practice recommendations ATA = American Thyroid Association; LBP = local best practice; FNA = fine-needle aspiration Adapted from the Princess Margaret Cancer Centre Clinical Practice Guidelines [10,21]

Sonographic Pattern	Risk of Malignancy	ATA Recommendations	LBP Recommendations
Benign	< 1%	No biopsy	FNA at > 4 cm
Very Low Risk	< 3%	Consider at > 2 cm	FNA at > 4 cm
Low Risk	5 - 10%	FNA at > 1.5 cm	FNA at > 2 cm
Intermediate	10 - 20%	FNA at > 1 cm	FNA at > 2 cm
High Risk	70 - 90%	FNA at > 1 cm	FNA at > 1 cm

All thyroid nodules that were sampled between January 1, 2018, and December 31, 2018, were reclassified based on their sonographic morphology and maximal size using the ACR TI-RADS algorithm with structured reporting similar to the Cancer Care Ontario (CCO) thyroid ultrasound reporting template and blinded from the cytopathology results [24]. This was performed to explore any impact ACR TI-RADS would have on the FNA rate at Bluewater Health. Cohen’s kappa was calculated at 0.56 correlating to 93% inter-rater reliability. Any discrepancy was rectified by consensus.

Starting from March 1, 2019, all thyroid US assessments and management recommendations were standardized to the ACR TI-RADS guidelines.

Statistics

Microsoft Excel (Microsoft Corporation, Redmond, WA) was used for data tabulation. Categorical data were described as counts. Statistical analysis was performed using GraphPad Prism (GraphPad LLC, San Diego, California). The z-score test for two population proportions was used to determine the level of significance between population characteristics for categorical data. A two-tailed t-test was used to compare normally distributed data to determine statistical significance. p≤0.05 was deemed statistically significant.

## Results

Data related to the study population are presented in Table 2. Briefly, the local best practice cohort (January 1, 2018, to December 31, 2018) comprised 57 thyroid nodules in 52 patients (mean age 66 ± 12; 40 Women). The ACR TI-RADS cohort (March 1, 2019, to March 31, 2020) comprised 57 thyroid nodules in 52 patients (mean age 61 ± 16; 41 Women). There were no statistical differences with respect to age, gender, or thyroid nodule location.

**Table 2:** Study Population ACR TI-RADS = American College of Radiology Thyroid Imaging Reporting and Data System; LBP = local best practice

	LBP Cohort	ACR TI-RADS Cohort	P-value
Patients	52	52	-
Thyroid nodules	57	57	-
Women	40	40	0.86
Age (mean ± range)	67 ± 12	62 ± 17	0.09
Location			
Right lobe	31	33	0.70
Left lobe	24	24	1
Isthmus	2	0	0.15

Thyroid nodules sampled between January 1, 2018, and December 31, 2018, were reclassified according to the ACR TI-RADS guidelines and presented in Table 3 with Bethesda System correlation. Thirty out of 57 thyroid nodules would have been followed using the ACR TI-RADS guidelines (TI-RADS 3 or less), and all of them were benign by pathologic assessment. Twenty-six out of 57 thyroid nodules would have required tissue sampling and all of them were TI-RADS 4 or 5 lesions. One thyroid nodule was categorized as benign (TI-RADS 2) with no follow-up or FNA recommendation.

**Table 3:** Reclassified LBP Cohort (January 2018 - December 2018) using ACR TI-RADS with Bethesda System correlation ACR TI-RADS = American College of Radiology Thyroid Imaging Reporting and Data System

ACR TI-RADS Classification	Bethesda System Results						
	1	2	3	4	5	6	Total
2	1	0	0	0	0	0	1
3	6	15	3	0	0	0	24
4	4	14	4	0	0	0	22
5	3	6	1	0	0	0	10
Total	14	35	8	0	0	0	57

Thyroid nodules sampled between March 1, 2019, and March 31, 2020, according to the ACR TI-RADS guidelines are presented in Table 4 with Bethesda System correlation. There were significantly more thyroid nodules with Bethesda III or above (malignant risk >10%) (21 in the ACR TI-RADS cohort versus 8 in the local best practice cohort, p = 0.005) and Bethesda IV or above (malignant risk > 25%) (7 in the ACR TI-RADS cohort versus 0 in the local best practice cohort, p = 0.006). There were similar rates of non-diagnostic/unsatisfactory samples (17.5% for the ACR TI-RADS group versus 24.6% for the local best practice group, p = 0.36).

**Table 4:** ACR TI-RADS cohort (March 2019 - March 2020) with Bethesda System correlation ACR TI-RADS = American College of Radiology Thyroid Imaging Reporting and Data System

ACR TI-RADS Classification	Bethesda System Results						
	1	2	3	4	5	6	Total
3	0	3	0	1	0	0	4
4	5	12	5	3	2	0	27
5	5	11	9	1	0	0	26
Total	10	26	14	5	2	0	57

## Discussion

Multiple thyroid nodule classification systems were developed to minimize the number of unnecessary FNAs while maintaining reasonable to high negative predictive values. ACR TI-RADS has been validated and shown to outperform the other classification systems in minimizing unnecessary FNAs with negative predictive values as low as 2.2% [15-20]. Starting from March 2019, Bluewater Health has transitioned from utilizing the LBP guidelines, which were largely based on the ATA classification system, to the ACR TI-RADS criteria for FNA recommendation.

Our results show a dramatic decrease in the number of unnecessary FNAs if the ACR TI-RADS guidelines were implemented from January to December 2018. Thirty (52.6%) of the previously sampled thyroid nodules using the LBP guidelines would have been followed as per ACR TI-RADS. This is a considerable amount given the amount of resources, costs, and patient anxiety that could have been averted. Our results also show that higher ACR TI-RADS corresponds to higher Bethesda grades. There were significantly more thyroid nodules with Bethesda III (malignant risk > 10%) in the ACR TI-RADS cohort versus the LBP cohort (21 versus 8, respectively, p = 0.005), as well as Bethesda IV or above (7 versus 0, respectively, p = 0.006). These findings are consistent with the literature demonstrating that ACR TI-RADS is reliable, specific, and outperforms the other classification systems in reducing unnecessary FNAs [15-20].

There is still room for improvement for ACR TI-RADS. Forty-five point six percent (45.6%) to 61.6% of the sampled thyroid nodules as per ACR TI-RADS showed benign cytology. Interobserver variability among radiologists and sonographers is an important consideration to improve TI-RADS performance. In a retrospective analysis of 127 nodules using ACR TI-RADS, Sahli et al. (2019) showed that while TI-RADS interobserver variability was fair (0.6 to 0.74), shape and margin criteria were the biggest sources of disagreement (poor; 0.359 and 0.192, respectively) [25]. Interestingly, in a separate study exploring sonographer performance and interobserver variability, Wildman-Tobriner et al. (2020) showed that sonographers also struggle with margins. Compared to the other sonographic criteria, shape and margin are scored either 0 or 3, and 0, 2, or 3, respectively. Differences in opinion with respect to shape and margin can certainly have a big impact on the overall TI-RADS score and, ultimately, whether a patient will need FNA or not [26].

A study suggested that decreasing the point assignment for punctate echogenic foci, particularly for mixed solid and cystic thyroid nodules, can also reduce the number of benign nodules [27]. Teefey et al. (2021) showed that of 287 mixed thyroid nodules, reducing the points assigned to punctate echogenic foci from 3 to 1 caused the overall TI-RADS score to change for 198 mixed nodules. Forty-four (44) benign nodules would not have been sampled. Although seven carcinomas would not have been sampled as well, six of them would have received follow-up. If the points assigned were changed from 3 to 2, eight benign nodules would not have been sampled and three carcinomas would have been followed instead of sampled [27].

Shape is currently a binary criterion in ACR TI-RADS (0 for wider-than-tall and 3 for taller-than wide). Grani et al. (2020) showed that applying a more specific AP/T ratio ≥1.2 would decrease unnecessary FNAs by up to 58.2% without a negative impact on sensitivity or diagnostic odds ratio [28].

Real-time tissue elastography can also improve the diagnostic performance of ACR TI-RADS [29-30]. Pei et al. (2020) showed that the elasticity score of real-time elastography and the malignant risk stratification of TI-RADS showed a strong correlation, particularly in the size intervals of 0.5 < D ≤ 1.0 cm, 1.0 < D ≤ 2.0 cm, and 2.0 < D ≤ 2.5 cm (r = 0.768, 0.711, and 0.743, respectively). The diagnostic performance of real-time tissue elastography in combination with ACR TI-RADS was consistently better than elastography or TI-RADS alone (p<0.001) [29].

The limitations of this study include the inherent biases of retrospective analysis, small sample size, as well as interobserver variability for both sonographers and radiologists.

## Conclusions

ACR TI-RADS significantly reduces the number of unnecessary thyroid FNAs compared to local best practice guidelines. A review of the literature suggests that further modifications to ACR TI-RADS may be helpful to improve overall diagnostic performance.
